# Burst agitation rate promotes sustained semicontinuous cultivation of filamentous fungi in stirred tank reactors

**DOI:** 10.1007/s00253-025-13579-y

**Published:** 2025-08-18

**Authors:** Conor Ó Lochlainn, Federico Cerrone, Kevin E. O’Connor

**Affiliations:** 1 School of Biomolecular and Biomedical Sciences, University College Dublin, Belfield Campus, Dublin, Ireland; 2 School of Biotechnology, Dublin City University, Glasnevin Campus, Dublin, Ireland; 3 BiOrbic Bioeconomy Research Centre, O’Brien Centre for Science (Science East) University College Dublin, Belfield Campus, Dublin, Ireland; 4 Bioplastech Ltd NovaUCD, Belfield Innovation Park, University College Dublin, Dublin, Ireland

**Keywords:** Bioactives, Biomass productivity, Filamentous fungi, High stirrer (speed), STR cultivation

## Abstract

**Abstract:**

Edible filamentous fungi *L. edodes* (shiitake mushrooms) were cultivated in submerged fermentation in stirred tank bioreactors (STR) both in batch and semicontinuous cultivation in a corn steep liquor (CSL) medium. The adjustment of a combination of constant impeller agitation speed, a short duration of a high-speed agitation (burst), and the frequency of bursts improved biomass (cell dry weight (CDW) titre from 1.75 to 4.95 g/L in a 96-h batch cultivation. These bioreactor process conditions were applied to a semicontinuous culture strategy to produce similar biomass density at a dilution rate of 0.02 h^−1^ for up to 10 days without washout over the duration of the fermentation. An increase in the dilution rate above 0.02 h^−1^ resulted in washout of *L. edodes* over time. Using a richer growth medium through the addition of malt extract, peptone, and molasses allowed *L. edodes* to grow to 4.7 g/L at a dilution rate of 0.025 h^−1^ without washout. The maximum biomass productivity (396 mg CDW/h) of the semicontinuous cultivation (*D* = 0.02 h^−1^) was 1.9-fold higher than the batch cultivation 206 mg CDW/hour. Use of the richer growth medium at *D* = 0.025 h^−1^ improved biomass productivity further to 470 mg/h. Glucans, known bioactives, were present in the fungal biomass at a maximum of 14% of the cell dry weight (CDW) with b-glucans predominating over a-glucans.

**Key points:**

• *Short, high-speed impeller bursts homogenise mycelia preventing bioreactor clogging.*

• *Semicontinuous fermentation at D of 0.02 h−1 generated 5.1 g/L of fungal biomass.*

• *Cultivation of fungal biomass in STR resulted in biomass with 14% of total glucans.*

**Supplementary Information:**

The online version contains supplementary material available at 10.1007/s00253-025-13579-y.

## Introduction

Filamentous fungi organised as spreading mycelia are adapted to forest, grasslands, and tundra soil ecosystems. They spread within the soil particles and often are in symbiosis with Gymnosperms and Angiosperms, facilitating nutrient recycling and uptake and ultimately contributing to ecosystem services. Filamentous fungi of the *Basidiomycota* division (*Agaricomycetes* class) form interconnecting networks of mycelia with a sophisticated reproductive regulation (Kües, 2015); Coelho et al. 2017) that originates fruiting bodies (mushrooms) as a means of dispersion of haploid spores when karyogamy and meiosis occur. These fungi synthesise polysaccharides that have been demonstrated to have immunomodulatory and therapeutic properties in humans (Municio et al. 2013), namely, glucans (α and β). There are also tissue-specific immunomodulatory effects linked to the branching and molecular weight of the β-glucans. Submerged cultivation of filamentous fungi in laboratory settings can increase the volumetric productivity of fungal biomass from weeks (solid substrate fermentation) to days. Within this controlled environment, the fungi will not develop the characteristic mushroom morphology (fruiting body cap) but will grow as a collection of mycelia aggregates (pellets). The control of the mycelia morphology is of paramount importance to avoid clogging of the bioreactor. This is of particular importance when growing fungi in a continuous culture. The peculiar metabolism of filamentous fungi will promote adhesion to solid surfaces and therefore hamper a proper homogeneous distribution of nutrients (carbon sources and dissolved oxygen) (Cairns et al. 2019; Waldherr et al. 2023). Airlift reactors avoid the shear stress of stirred tank bioreactors, but the level of fungal adhesion to surfaces can be high, resulting in poor circulation within the airlift. Different bioreactor designs can be envisaged to address adhesion (Cerrone and O'Connor, 2025), but a careful balance is needed between overcoming adhesion and inducing shear stress. Stirred tank reactors (STR) are particularly prone to induce shear stress in sensitive microorganisms; β-glucans impaired filamentous strains were reported to improve both hyphal dispersion and oxygen uptake rate (OUR) in STR. This aspect can be rewarded with increased protein and biomass yields, but it has been linked to a more pronounced shear stress (Susukida et al. 2025). The object of this study was to obtain a novel strategy to control adhesion while maximising the fungal biomass production in batch and semicontinuous cultivations in a STR.

## Materials and methods

### Basidiomycota strains

The fungal strain *L. edodes* (M3102) was purchased from Mycelia®, Deinze, Belgium. The stock culture was maintained on agar slants and sub-cultured once a month. The agar slant media consisted of the following reagents (bought from Sigma-Aldrich, Dublin, Ireland): 5 g/L of yeast extract (YE), 3 g/L of malt extract (ME), 2 g/L of soy peptone (SP), 2 g/L of glucose, and 15 g/L of agar. The pH of the media was 6.9.

### Conical flasks growth and inoculum preparation

To prepare the inoculum for the submerged cultivation in conical flasks, the following procedure was used: the selected fungal strain was grown by adding vegetatively growing and cryopreserved malt agar (MA) plates and incubated at 28 °C for 5 days. Five agar cubes (about 4 mm in diameter) were cut from the agar at the leading edge of the mycelium and placed into 250-mL conical flasks containing 100 mL of media that consisted of 15 mL/L of corn-steep liquor, CSL (Santa Cruz Biotechnology, Heidelberg, Germany) 10 g/L of glucose (Sigma-Aldrich, Dublin, Ireland), and 3.3 g/L of KH_2_PO_4_ (Fisher Scientific, Dublin, Ireland); the flasks were kept at a pH of 5.6. These cubes were then homogenised using an IKA T18 digital ULTRA TURRAX® homogeniser (IKA, Staufen, Germany), the tip of which was sterilised by autoclave. The flasks were placed in a New Brunswick Scientific Innova® (Eppendorf AG, Hamburg, Germany) 44 rotary shaker and shaken at 150 RPM at 25 °C for 7 days, which resulted in the growth of fungal pellets. The fungal biomass was maintained in liquid media stocks on a rotary shaker table shaking at 150 RPM, with the temperature maintained at 25° C. The culture was refreshed every 7 days. The stocks consisted of 100 mL of media in 250-mL flasks. The stocks were refreshed by homogenising the contents of one flask after 7 days of growth and pipetting 5 mL of the homogenised suspension to inoculate another 250-mL flask, which was subsequently shaken in a rotary shaker at 150 RPM for 7 days at 25 °C. This process was repeated every 7 days to maintain stocks. An 800-mL inoculation flask (to be used in the stirred tank reactor (STR) was prepared in the following manner. Two 7-day-old 250-mL flasks with a fungal strain containing 100 mL growth media (15 mL/L CSL, 10 g/L glucose, 3.3 g/L KH_2_PO_4_) were homogenised with a homogeniser and were each transferred to two 2-L flasks containing 700 mL of 15 mL/L CSL, 10 g/L glucose, and 3.3 g/L KH_2_PO_4_, making the total volume for each flask 800 mL. After 10 days, the biomass of one flask was drained through a sieve. The contents of the other flask and the drained biomass were transferred to a 1-L Duran bottle. Due to the fact that some evaporation had occurred during flask growth, the total resulting volume of medium and pellets in the Duran bottle was roughly 800 mL. The biomass of this bottle was homogenised for 30 s. Following the homogenisation, the contents of the Duran bottle were transferred to a fermenter (bioreactor) using a peristaltic pump connected to the Biostat C (Sartorius™ AG, Göttingen, Germany) stirred tank reactor (STR) using a Watson Marlow (Spirax Group PLC, Cheltenham, UK) peristaltic pump.

### Cryopreservation of the fungal strain

*L. edodes* was grown in cultivation flasks in the above-mentioned media for 5 days, until a homogenous population of pellets developed an approximate size of 3 mm. Pelletised aggregates cultivated in flasks were homogenised in sterile conditions using an IKA® T18 UltraTurrax® (IKA, Staufen, Germany) submergible homogeniser, equipped with a stainless steel S18 N 10G accessory that facilitates dispersion of the mycelia aggregates. After homogenisation, 100 μL of the culture was transferred into sterile cryovials already containing 450 μL of sterile glycerol (60% v/v solution) and 450 μL of sterile sucrose (30% w/v solution) for a final volume of 1 mL. The cryovials were kept in an ice bath at 0 °C for 5 min before being transferred into a − 70 °C Revco® (Thermo Scientific, Dublin, Ireland) Elite plus® freezer.

In order to mix the mycelia with the cryoprotectants, the following steps were taken: firstly, fungal pellets that had been grown in 100 mL of 15 mL/L CSL, 10 g/L glucose, and 3.3 g/L KH_2_PO_4_ media in 250-mL flasks at 250 RPM were homogenised. Fifty microliters of the homogenised pellets and spent media was transferred to a cryovial. For cryovials (i) and (ii), 950 µL of the cryoprotectants was added. For the other cryovials in which each cryoprotectant was added along with skimmed milk, 475 µL of the cryoprotectant was added along with 475 µL skimmed milk. The cryovials had a total volume of 1 mL. The vials were put into an ice bath for 5 min and then transferred to a − 80 °C freezer.

In order to test for the viability of the strains after cryopreservation, the vials were thawed and the contents transferred onto an agar plate (30 g/L ME, 3 g/L SP, 15 g/L agar) and their growth analysed.

### Batch cultivation in stirred tank reactor (STR)

A Biostat-C (Sartorius™ AG, Göttingen, Germany) 5-L total working volume vessel was chosen for batch fermentation. Dissolved oxygen (DO) and pH were constantly monitored for the duration of the growth period using online probes. In particular, DO was maintained using a constant flow of air (provided with an air compressor) of 1 vvm (4 slpm); a limit of 40% of oxygen saturation in the media was maintained as a reference point; the background agitation rate was set at 400 RPM, which equates to an impeller tip speed of 1.3 m/s. The agitation rate was modified by sets of burst speed increases for specific time frames. If ever the DO limit of 40% was reached, an increase in agitation rate (RPM) was also set up to be activated as a cascade control. The pH was controlled automatically at 5.6 ± 0.1 by the addition of 20% v/v NH_4_OH solution (Sigma-Aldrich, Dublin Ireland) or 15% v/v H_2_SO_4_ (Sigma-Aldrich, Dublin Ireland); foaming was controlled by the automatic addition of antifoam (polypropylene glycol P2000, Sigma-Aldrich, Dublin Ireland) when required. A laptop computer was used to remotely record data every 5 s. The accumulated data were recorded into BioPAT® MFCS SCADA fermentation software (Sartorius™ AG, Göttingen, Germany). A homogenised inoculum of 800 mL was used for a 4-L working volume. The temperature of the fermenter was set to 25 °C.

### Semicontinuous cultivations in stirred tank reactor (STR)

A Biostat-C (Sartorius™ AG, Göttingen, Germany) 5-L total working volume vessel was also chosen for semicontinuous fermentation studies. Dissolved oxygen (DO) and pH were constantly monitored for the duration of the growth period using online probes. In particular, DO was maintained using a constant flow of air (provided with an air compressor) of 1 vvm (4 slpm); a limit of 40% of oxygen saturation into the media was maintained as a reference point; the background agitation rate was set at 400 RPM, which equates to an impeller tip speed of 1.3 m/s. The agitation rate was modified by sets of burst speed increases for specific time frames. If ever the DO limit of 40% was reached, an increase in agitation rate (RPM) was also set up to be activated as a cascade control. The pH was controlled automatically at 5.6 ± 0.1 by the addition of 20% v/v NH_4_OH solution (Sigma-Aldrich, Dublin, Ireland) or 15% v/v H_2_SO_4_ (Sigma-Aldrich, Dublin, Ireland); foaming was controlled by the automatic addition of antifoam (polypropylene glycol P2000, Sigma-Aldrich, Dublin, Ireland) when required. A laptop computer was used to remotely record data every 5 s. The accumulated data were recorded into BioPAT® MFCS SCADA fermentation software (Sartorius™ AG, Göttingen, Germany). A homogenised inoculum of 800 mL was used for a 4-L working volume. The temperature of the fermenter was set to 25 °C. The semicontinuous regime was obtained by the use of a continuous process modulus (CPM) manufactured by Bionet™ (Alamo de Murcia, Spain) externally controlled by Rosita™, proprietary software of the same company. The CPM is provided with two peristaltic pumps with different heads, which can either work in continuous or semicontinuous mode for the addition of fresh medium or the withdrawal of spent medium. The head of the pump for the media addition works in a range of 17 to 300 mL/min, while the head of the pump for the media withdrawal works in a range of 368 to 1760 mL/min.

### Analytical procedures

After harvesting, the pelletised fungal biomass was filtered through a 90-µm membrane filter. The filter was made of stainless steel, and its diameter was 200 mm. The filtration was carried out by gravity and at room temperature. The mycelium was then resuspended in water for 2 min to clear away any excess media and was filtered again using the same method. The amount of water that the mycelia were resuspended in was equal to the quantity of media from which the mycelia were obtained. The harvested pelletised fungal biomass was frozen into a − 70 °C Revco® (Thermo Scientific, Dublin, Ireland) Elite plus® freezer, on bulk trays designed for a Labconco™, (Kansas City, MO, USA) freeze dryer. After freezing, the mycelia were transferred to the Labconco™ freeze-dryer, and the freeze-dryer created a near-vacuum of 0.1 mbar, which caused the water present in fungal samples to evaporate by sublimation. Freeze drying lasted for 72 h. After freeze-drying, the biomass was then weighed using an Ohaus™ Explorer weighing scale (Ohaus™, Nänikon, Switzerland), and the value was used to determine the concentration (g/L) of mycelium harvested from shake flasks and bioreactors. Glucose and analyses of other monosaccharides were detected by using a Shimadzu®, (Duisburg, Germany) HPLC equipment provided with a RID detector. The used mobile phase was a 0.014N H_2_SO_4_ solution, prepared in Milli-Q water, subsequently vacuum filtered with a 0.2 μm pore size filter; this mobile phase was used in an isocratic fashion. The column used was an Aminex HPX-87H (BioRad®, Watford, England) with a solid polymeric matrix composed of polystyrene divinylbenzene. The flow rate of the mobile phase was 0.55 mL/min. α- and β-glucans were detected by an enzymatic-based colorimetric method using a specific yeast/fungi glucan detection kit (Megazyme®, Wicklow, Ireland) following the company provided protocol.

### Statistical analysis of results

The averaged values of CDW obtained varying the amount of bursts speed per day, or varying the impeller constant speed or varying the impeller burst speed are tabulated in Tables 1, 2 and 3, respectively, and were obtained by triplicate experiments. The achieved values of fungal CDW in batch and semicontinuous fermentations were also obtained by triplicate independent experiments. Analyses of variance (specifically, one-way ANOVA) of the mean values of CDW obtained with different impeller conditions (batch fermentations) and with different dilution rates (semicontinuous fermentations) were performed to determine a statistical difference across results, and they were performed using the corresponding function in Excel™; *T*-test and ad hoc Tukey test were performed on the ANOVA results to allow both pairwise and total comparisons of them; *T*-test and Tukey’s test were also performed using the corresponding function in Excel™.

**Table 1:** Impact of the number of impeller tip speed bursts on biomass and glucan content of *L. edodes* cells grown in batch culture

Bursts per day*	CDW (g/L)	Total glucan (%)	α-glucan (%)	β-glucan (%)
1	1.75 ± 0.08	12.15 ± 1.73	2.25 ± 0.51	9.9 ± 2.24
2	2.54 ± 0.5	12.72 ± 1.06	1.43 ± 0.6	11.28 ± 0.47
4	4.95 ± 0.31	13.23 ± 0.26	1.87 ± 0.25	11.36 ± 0.01
6	4.07 ± 0.19	14.36 ± 0.02	2.18 ± 0.71	12.19 ± 0.73

*Burst tip speed of impeller = 4.9 m/s. Constant impeller tip speed 1.3 m/s

**Table 2:** The CDW and glucan content of *L. edodes* grown in a stirred tank bioreactor with various “constant impeller” tip speeds

Impeller constant speed (tip speed in m/s)*	CDW (g/L)	Total glucan (%)	α-glucan (%)	β-glucan (%)
200 (0.7)	4.34 ± 0.5	11.07 ± 0.48	1.49 ± 0.25	9.58 ± 0.23
400 (1.3)	4.95 ± 0.31	13.23 ± 0.26	1.87 ± 0.25	11.36 ± 0.01
600 (2)	4.28 ± 0.5	15.67 ± 0.7	1.51 ± 0.94	14.16 ± 0.23

In addition to the constant impeller speed a high speed impeller burst with a tip speed of 4.9 m/s was applied for 30 s every 6 h. 0.7 m/s = 200 RPM, 1.3 m/s = 400 RPM, 2 m/s = 600 RPM

**Table 3:** The effect of impeller tip speed variation in a stir tank reactor (STR) on the growth and glucan content of *L. edodes* when grown in batch mode

Impeller burst speed (tip speed in m/s)*	CDW (g/L)	Total glucan (%)	α-glucan (%)	β-glucan (%)
1500 (4.9)	4.95 ± 0.31	13.23 ± 0.26	1.87 ± 0.25	11.36 ± 0.01
1200 (3.9)	4.76 ± 0.42	13.3 ± 0.53	2.19 ± 0.36	11.11 ± 0.17
1000 (3.2)	4.22 ± 0.24	13.15 ± 1.24	2.86 ± 0.76	9.96 ± 1.74
800 (2.6)	4.27 ± 0.23	14.68 ± 1.24	2.74 ± 0.19	11.94 ± 1.05

*Frequency of burst = 1 every 6 h. *4.9 m/s = 1500 RPM, 3.9 m/s = 1200 RPM, 3.2 m/s = 1000 RPM, 2.6 m/s = 800 RPM. Constant impeller tip speed was set at 1.3 m/s (400 RPM)

## Results

### Batch cultivation of *L. edodes* in STR with a burst agitation rate control

In a stirred tank bioreactor, the agitation rate (in RPM) is directly correlated to the dissolved oxygen concentration. A high agitation rate is able to increase the surface area of the air bubbles and therefore increase the oxygen transfer, but this also comes with higher shear stress (Meyer et al. 2021). *L. edodes* was grown in batch mode in a 5 L (4 L working volume) STR. The growth media used consisted of 15 mL/L of corn steep liquor (CSL), 10 g/L glucose and 3.3 g/L potassium phosphate (KH_2_PO_4_) (Cerrone et al. 2024). During the initial hours of incubation, the impellers prevented clump formation in the submerged area of the fermenter; however, clumps formed at the surface of the media and in the headspace of the fermenter because of fungal pellets being thrown up by the impellers and adhering to the glass and metal frame. The formation of large clumps is known to impede oxygen uptake into the inner layers of the fungal pellet (Espinosa-Ortiz et al. 2016) resulting in reduced metabolic activity. Fungal pellets began to form shortly after inoculation and slowly grow larger over time into mycelia clumps; the resulting formation of mycelia clumps on the glassware, probes, and impeller causes the clogging of the bioreactor. While high agitation of the impeller (rpm) is linked to shear stress and reduced growth rate of filamentous fungi, it can also reduce the formation of mycelia clumps; we wanted to explore the balance between reducing mycelial clump formation and promoting the growth of the fungus. The optimisation of the reduction of mycelia clumps formation was attempted with the variation of a combination of various factors, i.e., short duration increases in impeller speed (bursts), frequency of the bursts (bursts per 24-h period), and constant impeller speed (Tables 1, 2, and 3).

At a constant impeller speed of 400 RPM (tip speed of 1.3 m/s), various “Impeller burst” speeds were tested to measure the impact on fungal biomass productivity. The best burst speed is considered the one which produces the highest fungal biomass (cell dry weight (CDW)), the highest total glucans (% of CDW), and effectively reduces mycelia clumping inside the bioreactor. The impeller tip speed was increased from 1.3 m/s (400 RPM) to 4.9 m/s (1500 RPM) for 30 s and then returned to the steady state impeller speed of 1.3 m/s. The mycelial clumps were broken up by the impellers once submerged in the rising and fast-moving liquid media in the vessel. One “burst” was undertaken every 24 h to remove the clumps that had formed. The resulting biomass of this fermentation was 1.75 ± 0.08 g/L (Table 1). The total glucan content as a percentage of cell dry weight was 12.15 ± 1.73%, with the β-glucan concentration accounting for over 81% of the total glucans present in the cell (Table 1). Mycelial clumps would start to form a few hours after the burst had occurred, which meant that the mycelial clumps would exist for most of the 24-h period. Due to this, burst frequency was increased to once every 12 h for subsequent fermentations. This resulted in a 1.45-fold increase in the biomass concentration (2.54 ± 0.5 g/L) (Table 1). The glucan content of the 2-bursts per day fermentation was unchanged (Table 1). Increasing the frequency of bursts per day further (4/day) was rewarded by a further increase of biomass concentration (4.95 g/L), but a further increase (6/day) caused a decrease of fungal biomass concentration (4.07 g/L) (Table 1). This moderate increased burst regime likely increased the biomass production in the fermentation by reducing the amount of time that there was less active or inactive clumping-biomass, but a more pronounced increase induces likely shear stress damage on the mycelia population, hindering further growth. Statistical analyses of these results identified that all the bursts per day combinations achieved statistically significant differences as it regards CDW; glucans % differences were statistically different only when comparing *T*-test values of 1 vs. 6 or 2 vs. 6 bursts per day (Supplemental Table 1).

Having determined the effect of burst speed, the experimental focus switched to the effect of constant tip speed or RPM of the rotor in combination with a set burst speed (1500 RPM) for 30 s and four times per day. At constant RPMs that were lower and higher than 400 RPM (200 RPM (0.7 m/s) and 600 RPM (2 m/s)), a decreased concentration of fungal biomass was obtained (4.34 g CDW/L and 4.28 g CDW/L, respectively) compared with 4.95 g CDW/L at 400 RPM (1.3 m/s). A slightly higher percentage of total glucans was obtained at a constant impeller speed of 600 RPM (2 m/s) (Table 2). Despite the differences in CDW, by changing the constant impeller speed, no statistically significant differences were obtained. On the other hand, the percentage of glucans at all the different constant impeller speeds demonstrated to be statistically significant (Supplemental Table 1).

Initial burst speed (duration 30 s) was tested at 1500 RPM (tip speed of 4.9 m/s); this impeller speed implies high energy requirements, so we also wished to determine if lower tip speeds could equally achieve mycelial clump breakage and allow favourable fungal growth. Burst speeds of 1200 RPM (tip speed of 3.9 m/s), 1000 RPM (tip speed of 3.2 m/s), and 800 RPM (tip speed of 2.6 m/s) resulted in similar fungal biomass (Table 3). The average CDW was 4.54 g/L, while the average of the total glucans was 13.59% (Table 3). This indicated that 800 RPM for 30 s was sufficient to break up mycelial clumps; therefore, both lower and higher speeds (800 vs. 1500 RPM) did not adversely affect the fungal growth. One-way ANOVA analysis of the CDW results regarding the impeller burst speed proved to be statistically significant (*P* value = 0.03, with a F-stat = 3.95 and F-crit = 3.49), while a subsequent pairwise comparison of the values of the *T*-test proved to be partially statistically significant (in particular for 1500 vs. 1000, 1500 vs. 800 and 1200 vs. 1000 RPM, respectively). The statistically significant difference (as it regards % of glucans) with the variation of the impeller burst speed was null.

### Semicontinuous cultivation of *L. edodes* in STR with a burst agitation rate control

With the objective of increasing the fungal biomass productivity in STR cultivation, we decided to examine a strategy of continuous cultivation. In this study, *L. edodes* underwent a range of semicontinuous cultivations at different dilution rates (D = 0.02, 0.025, 0.03 and 0.04 h^−1^), applying the most successful combination of “constant agitation” and impeller “bursts” from the batch experiments (constant 1.3 m/s tip speed (400 RPM), bursts at 4.9 m/s (1500 RPM), and 4 bursts per day). The term semicontinuous is mentioned because the silicon tube to remove the fungal biomass has to be a wider bore than the tube feeding the medium into the bioreactor to allow removal of the fungal pellets from the STR. Thus, due to the design of the peristaltic pump head, it is not possible to have the inlet flow and the outlet flow continuously matching. The two-target flow rates (media addition and spent media/biomass withdraw) match in a cyclical fashion every given hour; this is technically considered a semicontinuous cultivation with a controlled dilution rate every hour. The liquid medium was supplied to the bioreactor at a slow rate, while the withdrawal of the spent media was removed at a fast rate for a limited period of time. For example, this means that for the first tested dilution rate (0.02 h^−1^ that equates to 80 mL for a 4L bioreactor), 80 mL of fresh media is added every hour (usually at a flow rate of 5.33 mL/min for 15 min), while 80 mL of spent media/biomass is withdrawn every hour (at a flow rate of 368 mL/min, for 13 s).

**Fig. 1:**
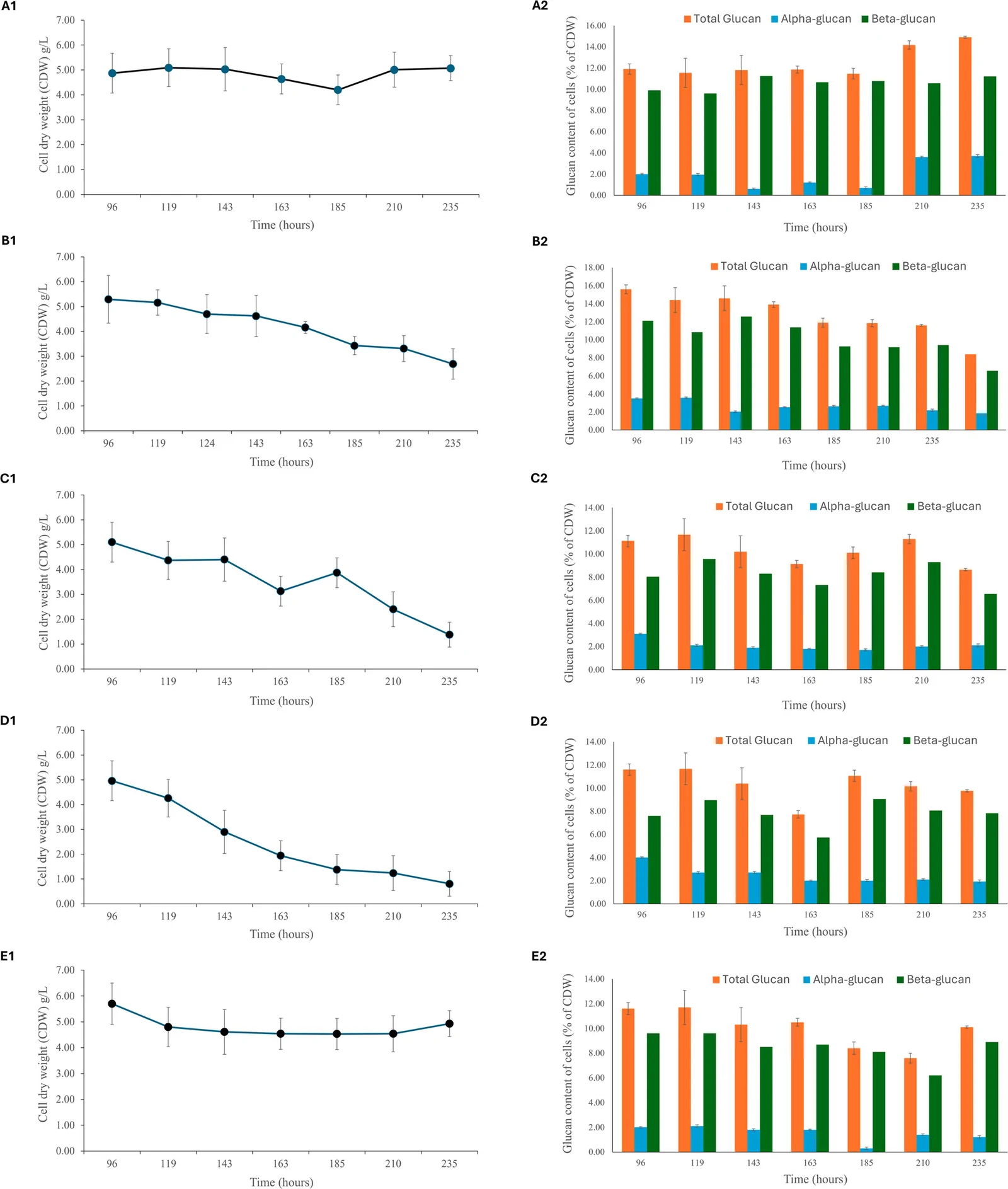
Cell dry weights (CDW) (A1 to E1, g/l) and glucan (A2 to E2, % CDW) of *L. edodes* grown in a semicontinuous culture at various dilution rates: 0.02 h^−1^ (**A**), 0.025 h^−1^ (**B**), 0.03 h^−1^ (**C**), 0.04 h^−1^ (**D**) using corn steep liquor (CSL) medium and at a dilution rate of 0.025 h^−1^ (**E**) using a rich medium in a stirred tank reactor (STR). The semicontinuous culture started after a batch growth of 96 h. Average CDW values (at the corresponding dilution rates) and average % content of each glucan were obtained by three independent experiments

At a dilution rate of 0.02 h^−1^, the fungal biomass reached a maximum cell dry weight (CDW) of 5.09 g/L (at 119 h) (Fig. 1, A1). Apart from a decrease in CDW between 163 and 185 h, *L. edodes* was able to maintain a mostly consistent CDW of approximately 5 g/L throughout the fermentation (Fig. 1, A1).

The total glucans concentration also remained mostly stable at around 12% of CDW (Fig. 1-A2). The β-glucan concentration ranged between 10 and 12% with b-glucan the major glucan type (8–10%) and the α-glucan varying between 2 and 4%. There was an increase in glucan content towards the end of the semicontinuous culture (after 210 h) which correlated with an increase in fungal biomass (Fig. 1, A1 and A2).

An increase in the dilution rate, if the biomass can be maintained, can increase volumetric productivity; however, we observed a decreasing biomass when the dilution rate was increased from 0.02 to 0.025, 0.03, and 0.04 h^−1^, respectively. On average, the CDW dropped by between 28% (at a D of 0.025 h^−1^) and by 65% (at a D of 0.04 h^−1^) (Fig. 1B–D). One-way ANOVA results showed statistically significant differences across the CDW trends at the four tested dilution rates (*P* value 0.0068, with *F*-stat = 5.14 vs. F-crit = 3.00); paired *T*-tests of the means confirmed that all the comparisons are statistically significant (at all the tested dilution rates), while for paired *T*-tests (that assume equal and unequal variances), all of them are statistically significant, except for CDWs at 0.025 vs. 0.03 h^−1^ and CDWs at 0.03 vs. 0.04 h^−1^ (Supplemental Table 1). Equally, the % of glucans showed statistically significant differences across the four tested dilution rates (*P* value 0.01, with *F*-stat = 4.23 vs. F-crit = 3.00), and paired *T*-tests comparisons showed statistically significant differences, except at 0.025 vs. 0.03 h^−1^ and at 0.03 vs. 0.04 h^−1^.

In order to increase the biomass, we focused on improving the growth medium. From a range of media design experiments (data not shown), an optimal medium for the production of *L. edodes* biomass was determined to be 22.5 g/L of malt extract (ME), 1 g/L of molasses, 2 mL/L of CSL, 6 g/L of glucose, 2 g/L of soy peptone (SP), 1 mL/L of Ramsay’s trace elements (Sun et al 2007), and 3.3 g/L of KH_2_PO_4_. This media allowed a 7% increase in *L. edodes* biomass production in shake flasks. We attempted a semicontinuous cultivation of *L. edodes* at a D of 0.025 h^−1^ with this growth media. A dilution rate of 0.025 h^−1^ was chosen because *L. edodes* washed out slowly at 0.025 h^−1^ with CSL media (Fig. 1, B1), and so we believed a richer media may help the cells to sustain growth at this dilution rate. Indeed, with the use of the optimal media, a more consistent CDW trend was obtained across the 235 h of semicontinuous cultivation. The average CDW was 4.8 g/L (Fig. 1, E1). One-way ANOVA comparison found no statistically significant differences between the CDW at a D of 0.025 h^−1^ (background medium) vs. 0.025 h^−1^ (richer medium) (*P* value = 0.1, with a *F*-stat = 3.07 and F-crit = 4.74), while the pairwise *T*-test comparison of the CDW at a D of 0.025 h^−1^ (background medium) vs. 0.025 h^−1^ (richer medium) gave a slightly statistically significant difference (*T*-stat = 2.14 vs. *T*-crit = 1.94 with a *P* value = 0.037). The average total glucan content of cells grown in both media did not differ more than 10%, but they showed a statistically significant decrease during the semicontinuous fermentation when the rich medium was used (Fig. 1, E2 and Supplemental Table 2).

The maximum biomass productivity (396 mg CDW/h) of the semicontinuous cultivation (*D* = 0.02 h^−1^) was 1.9-fold higher than the batch cultivation 206 mg CDW/h). The use of the rich growth medium at *D* = 0.025 h^−1^ improved biomass productivity further to 470 mg/h.

## Discussion

The submerged cultivation of filamentous fungi is a complex interlinkage of fluid dynamics and biological metabolism. Vegetative growth of filamentous fungi in submerged cultivation causes the formation of mycelia pellets of different diameters and densities, composed of aggregating hyphae. This aspect is directly linked to the volumetric productivity of the system (Veiter et al. 2018). Each individual hypha is made of three main type of cells (i.e., apical, subapical, and hyphal cells) (Nielsen, 1993); the growth of these mycelia can be modelled by using the concept of hyphal growth unit (HGU); the rationale behind this model implies that the actively growing cells are only the apical and subapical cells and that when an apical cell emerges from a subapical cell, it is considered a branching point of the mycelia. The growth rates of individual apical (a) and subapical (s) cells are described by a Monod model linearly correlated to the individual substrate uptake by the cell, where the growth rate of the hyphal (h) cell is zero [1].

1 $$\begin{array}{l}{\mu }_{a}={k}_{a}S/\left(S+{K}_{S}\right)\\ {\mu }_{s}={k}_{s}S/\left(S+{K}_{S}\right)\\ {\mu }_{h}=0\end{array}$$

Equation [1] terms: *μ*_a/s/h_ is the growth rate of apical/subapical or hyphal cells (in h^−1^); *k*_a/s_ is the half saturation constant for apical or subapical cells, respectively where μ/μ_MAX_ = 0.5; *S* is the concentration of substrate (in g/L).

The mass of a hyphal growth unit (*m*_hgu_) can be defined as the ratio of the total mass of the mycelia divided by the number of actively growing tips. The mass formation of each individual pellet is directly correlated to oxygen availability and is therefore paramount to allow dynamic controls that maximise the oxygen availability for the actively growing cells of the mycelia. The variation of the mechanical agitation is a strategy to increase oxygen availability to cells, but this aspect needs to be balanced with the negative consequences of shear stresses (Waldherr et al. 2023) considering the mechano-response of the hyphae; this last aspect was modelled by (Schrader et al. 2024) on individual pellets, by using discrete element method (DEM); these authors found that pellets with different morphologies (from flat to spherical) were created depending on the increasing hyphal branching angle and that the compression forces acting on a pellet were proportional to the hyphal fractal behaviour and inversely proportional to the change in hyphal orientation; according to Money (2025), the hyphal fitness and their mechano-response are constantly dictated by biophysical forces acting on the filamentous microorganisms. Submerged cultivation of filamentous fungi is characterised by a constant interplay of hydrodynamic forces that influence the hyphal growth and ultimately affects the yields of product formation; it is therefore paramount to dynamically control the agitation regime to maximise the biomass volumetric productivity, by increasing oxygen availability, reducing fungal biomass adhesion but also by reducing its shear stress.

In a batch fermentation, using a CSL-based medium, *L. edodes* achieved a volumetric productivity of 90.4 mg/L/h (2.17 g/L/day) which occurred between hour 48 and hour 72. Özdemir et al. (2017) reported a much lower biomass productivity for *L. edodes*, i.e., 2.72 mg/L/h in a 1 L STR with a working volume of 800 mL after 72 h at 24 °C; a similar result in biomass productivity was also found by Mancera-Martinez et al. (2023) where 4.3 mg/L/h was produced after 152 h in an 80 RPM agitated STR. Fukushima et al. (1993) reported a volumetric productivity of 37.5 (mg of dry weight of mycelium/L/h) in a continuous fermentation that was higher (twofold) than in a batch fermentation. These authors also reported a marked washout of fungal biomass above a dilution rate of 0.01 h^−1^ with an average halving of the CDW. While we observed washout at dilution rates above 0.02 h^−1^ with CSL medium, we did not encounter washout or CDW decrease of the fungal biomass, at a dilution rate of 0.02 h^−1^ (CSL medium) or 0.025 h^−1^ (rich medium) likely due to the agitation strategy.

Our volumetric productivity is 4.8-fold (batch) and sevenfold (semicontinuous) higher than the one found by Fukushima et al. (1993), and similarly, we saw a 2.9-fold increase in volumetric productivity between batch (90.4 mg/L/h) and semicontinuous (265 mg/L/h) phases in our study; our volumetric productivity is also 100-fold higher than that reported by Özdemir et al. (2017), confirming the big variability one can achieve in similar setup without optimised conditions.

A careful and dynamic control of different agitation rates regimes has been shown to have a profound effect on the total fungal biomass concentration and consequential volumetric productivity; to the best of our knowledge, no studies focused on this aspect for the batch and continuous cultivation of *L. edodes* in stirred tank bioreactors. Jung et al. (2008) found that lower constant impeller agitation rates (100 and 200 RPM) initially resulted in the production of small pellets for the fungus *H. erinaceus*, while higher agitation rates (300 and 400 RPM) initially resulted in the production of large pellets; this inverse correlation between pellet size and agitation rate is also observed by Mancera-Martinez et al. (2023) when cultivating *L. edodes* in STR. However, pulp mycelia formed as both fermentations progressed, which is thought to occur as a result of the collision of pellets with impellers or baffles. Waldherr et al. (2023) explored different turbine designs for the fragmentation of *A. niger* in STR and also saw a clear decrease in pellet size when the power input of the stirrer was increased. These researchers achieved a similar fungal biomass concentration for *A. niger* as the one achieved for *L. edodes* in this study. Bustamante et al. (2024) also experimented with turbine design in STR for the cultivation of filamentous fungi; they saw a clear advantage of using elephant-ear blades for both the increase of branched hyphae and for the improvement of the product (clavulanic acid) volumetric productivity but at the expense of the increase of both the broth viscosity and clumps formation; conversely, they saw a clear advantage in using Rushton-type blades through the increase of unbranched hyphae and the reduction of pellets and clumps formation; the biomass concentrations are not reported so their biomass volumetric productivity cannot be compared with our study.

Tamerler and Keshavarz (1999) reported that impellers in a bioreactor can damage fungal cells if the tip speed is too high. However, clumping of fungal biomass occurs within 24 h (Figure S1) and progresses with 3 to 4 days to a point that impedes media mixing and airflow in the bioreactor with *L. edodes*, hampering continuous cultivation. In the current study, we are demonstrating that the dynamic variation of the RPM, alternating between a constant agitation and a burst agitation, can result in good biomass growth and avoid clumping and heterogeneous distribution of fungal biomass in the bioreactor, thus prolonging the fermentation, which is advantageous in a continuous culture. The most effective combination was to have a strong burst agitation (800–1500 RPM) for 30 s, four times per day.

Fungal pellet morphology and aggregation is a complex phenomenon driven by multiple factors. Nair et al. (2016) identified a clear role not only of the hydrodynamic stress but also of the medium pH in dictating the resulting pellets morphology; an acidic pH would limit the ability to establish fungal pellets aggregation into clumps. Some researchers opted to strictly control the pH throughout the fermentation. Kim et al. (2002) and Rancaño et al. (2003) determined that a pH of 5 was the optimal pH, while they saw a fungal biomass decrease when the pH was uncontrolled; we did not control the pH in this study, allowing the physiological pH control to occur; having pH control could have increased the fungal biomass concentration but would also likely induce chances of fungal pellets adhesion and entrapment. Extending the duration of the continuous fermentation, having both a viable and homogenous population of fungal pellets, is a combined effort that requires managing both the hydrodynamic stress and the fungal metabolism. A combination of agitation regimes not only extended the duration of the STR-based cultivation but also showed the potential of using up to a D of 0.025 h^−1^ for *L. edodes* production. A clear statistical difference in the decrease of CDW production can be seen when the D is increased to suboptimal levels (above 0.025 h^−1^). This set of results is unprecedented in the scientific literature and could set the benchmark for studies of media design to increase further the D and achieve a sustainable fungal biomass production. This edible biological platform is essential for sustainable biobased production and nutrient recycling that are at the core of successful circular solutions.

## Conclusion

A careful combination of a background constant impeller regime (400 RPM) with 30-s bursts (at 1200–1500 RPM), every 4 h maximises the CDW production of *L. edodes*, cultivated in submerged cultivation in a STR. This dynamic control reduces the adhesion of the fungal biomass and extends the duration of semicontinuous fermentations when a suitable dilution rate is used (up to 0.025 h^−1^), improving the volumetric productivity and potentially allowing the extension of the fermentation further, by using the same process design strategy. A similar approach could be potentially envisaged for the submerged cultivation of other *Agaricomycetes*.

## Supplementary Information

Below is the link to the electronic supplementary material.

ESM 1 (DOCX 2.91 MB)

## Data Availability

The authors declare that the data supporting the findings of this study are available within the paper and its Supplementary Information files. Should any raw data files be needed in another format, they are available from the corresponding author upon reasonable request.
