# Supplementary material for: Burst agitation rate promotes sustained semicontinuous cultivation of filamentous fungi in stirred tank reactors
Source: Appl Microbiol Biotechnol. 2025 Aug 18;109:185. doi: 10.1007/s00253-025-13579-y (PMC12361324; doi:10.1007/s00253-025-13579-y)
Supplement: Supplementary file 1 [file 253_2025_13579_MOESM1_ESM.docx]

Applied Microbiology and Biotechnology – Supplemental material.

**Burst agitation rate promotes sustained semicontinuous cultivation of filamentous fungi in stirred tank reactors.**

Conor Ó Lochlainn^1,3^, Federico Cerrone^2,3,*^, Kevin O’Connor^1,3,4,**^

^1^School of Biomolecular and Biomedical Sciences University College Dublin, Belfield Campus Dublin, Ireland

^2^School of Biotechnology, Dublin City University, Glasnevin Campus Dublin, Ireland

^3^BiOrbic Bioeconomy Research Centre, O’Brien Centre for Science (Science East) University College Dublin, Belfield Campus Dublin, Ireland

^4^Bioplastech Ltd NovaUCD, Belfield Innovation Park, University College Dublin, Ireland

*[Federico.cerrone@dcu.ie](mailto:Federico.cerrone@dcu.ie) telephone and fax number +353(1)7006813 *[*Kevin.oconnor@ucd.ie](mailto:*Kevin.oconnor@ucd.ie), telephone and fax number +353(1)7162198

**Supplementary information**

**Figure S1.** (A) An STR fermentation wherein a large clump has formed in the headspace of the fermenter after 24 hours and (B) An STR fermentation after the removal of a clump using a burst (1500 rpm) of impeller speed for 30 seconds**.**

Supplementary Table 1

|  | **Bursts per day** | **T-test** | | | **ANOVA (one-way)** | | |
| --- | --- | --- | --- | --- | --- | --- | --- |
|  |  | **T-stat** | **T-crit** | **P-value** | **F-stat** | **F-crit** | **P-value** |
| **CDW** | 1 vs 2 | 2.51 | 2.13 | 0.03 | 59.09 | 4.06 | 0.000008 |
|  | 1 vs 4 | 17.4 | 2.13 | 0.00003 |  | | |
|  | 1 vs 6 | 19.0 | 2.13 | 0.00002 |  |  |  |
|  | 2 vs 4 | 6.77 | 2.13 | 0.001 |  |  |  |
|  | 2 vs 6 | 4.65 | 2.13 | 0.004 |  |  |  |
|  | **Constant impeller speed (RPM)** | **T-test** | | | **ANOVA (one-way)** | | |
|  |  | **T-stat** | **T-crit** | **P-value** | **F-stat** | **F-crit** | **P-value** |
|  | 200 vs 400 | 1.60 | 2.13 | 0.09 | 1.60 | 5.15 | 0.27 |
|  | 200 vs 600 | 0.11 | 2.13 | 0.45 |  | | |
|  | 400 vs 600 | 1.76 | 2.13 | 0.07 |  |  |  |
|  | **Impeller bursts speed (RPM)** | **T-test** | | | **ANOVA (one-way)** | | |
|  |  | **T-stat** | **T-crit** | **P-value** | **F-stat** | **F-crit** | **P-value** |
|  | 1500 vs 1200 | 0.61 | 1.94 | 0.27 | 3.95 | 3.49 | 0.03 |
|  | 1500 vs 1000 | 3.17 | 1.94 | 0.009 |  | | |
|  | 1500 vs 800 | 2.97 | 1.94 | 0.001 |  |  |  |
|  | 1200 vs 1000 | 1.94 | 1.94 | 0.05 |  |  |  |
|  | 1200 vs 800 | 1.75 | 1.94 | 0.06 |  |  |  |
|  | 1000 vs 800 | 0.24 | 1.94 | 0.41 |  |  |  |
|  | **Bursts per day** | **T-test** | | | **ANOVA (one-way)** | | |
|  |  | **T-stat** | **T-crit** | **P-value** | **F-stat** | **F-crit** | **P-value** |
| **% of Glucans** | 1 vs 2 | 0.48 | 2.13 | 0.32 | 2.52 | 4.06 | 0.13 |
|  | 1 vs 4 | 1.06 | 2.13 | 0.17 |  | | |
|  | 1 vs 6 | 2.21 | 2.13 | 0.04 |  |  |  |
|  | 2 vs 4 | 0.80 | 2.13 | 0.23 |  |  |  |
|  | 2 vs 6 | 2.66 | 2.13 | 0.028 |  |  |  |
|  | **Constant impeller speed (RPM)** | **T-test** | | | **ANOVA (one-way)** | | |
|  |  | **T-stat** | **T-crit** | **P-value** | **F-stat** | **F-crit** | **P-value** |
|  | 200 vs 400 | 6.87 | 2.13 | 0.001 | 64.3 | 5.15 | 0.00008 |
|  | 200 vs 600 | 5.90 | 2.13 | 0.002 |  |  |  |
|  | 400 vs 600 | 9.70 | 2.13 | 0.0003 |  |  |  |
|  | **Impeller bursts speed (RPM)** | **T-test** | | | **ANOVA (one-way)** | | |
|  |  | **T-stat** | **T-crit** | **P-value** | **F-stat** | **F-crit** | **P-value** |
|  | 1500 vs 1200 | 0.27 | 2.13 | 0.4 | 1.84 | 4.06 | 0.21 |
|  | 1500 vs 1000 | 0.1 | 2.13 | 0.45 |  | | |
|  | 1500 vs 800 | 1.98 | 2.13 | 0.06 |  |  |  |
|  | 1200 vs 1000 | 0.22 | 2.13 | 0.41 |  |  |  |
|  | 1200 vs 800 | 1.73 | 2.13 | 0.08 |  |  |  |
|  | 1000 vs 800 | 1.51 | 2.13 | 0.1 |  |  |  |

Supplementary Table 1. Analysis of variance (one-way ANOVA) and T-test with equal variance of the Cell Dry Weight (CDW) and % of glucans of *L. edodes* biomass, cultivated in stirred tank reactor (STR) by varying: the frequency of impeller bursts per day, the constant impeller speed and the impeller burst speed over a background constant impeller speed of 400 revolution per minute (RPM). Grey highlighted values are the statistically significant values. (when P-value < 0.05)

Supplementary Table 2

|  | **Dilution rate (h^-1^)** | **T-test** | | | **ANOVA (one-way)** | | | | |
| --- | --- | --- | --- | --- | --- | --- | --- | --- | --- |
|  |  | **T-stat** | **T-crit** | **P-value** | **F-stat** | | **F-crit** | **P-value** | |
| **CDW** | 0.02 vs 0.025 | 1.90 | 1.78 | 0.04 | 5.14 | | 3.00 | 0.006 | |
|  | 0.02 vs 0.03 | 2.62 | 1.78 | 0.01 |  | | | | |
|  | 0.02 vs 0.04 | 3.80 | 1.78 | 0.001 |  |  |  |  |  |
|  | 0.025 vs 0.03 | 0.92 | 1.78 | 0.18 |  |  |  |  |  |
|  | 0.025 vs 0.04 | 2.24 | 1.78 | 0.02 |  |  |  |  |  |
|  | 0.03 vs 0.04 | 1.31 | 1.78 | 0.11 |  |  |  |  |  |
|  | 0.025 (background medium) vs 0.025 (rich medium) | 2.14 | 1.94 | 0.037 | 3.07 | | 4.74 | 0.10 | |
|  | **Dilution rate (h^-1^)** | **T-test** | | | **ANOVA (one-way)** | | | | |
|  |  | **T-stat** | **T-crit** | **P-value** | **F-stat** | | **F-crit** | **P-value** | |
| **% of Glucans** | 0.02 vs 0.025 | 0.48 | 1.78 | 0.49 | 4.23 | | 3.00 | 0.01 | |
|  | 0.02 vs 0.03 | 3.25 | 1.78 | 0.003 |  | | | | |
|  | 0.02 vs 0.04 | 2.97 | 1.78 | 0.005 |  |  |  |  |  |
|  | 0.025 vs 0.03 | 2.23 | 1.78 | 0.02 |  |  |  |  |  |
|  | 0.025 vs 0.04 | 2.13 | 1.78 | 0.027 |  |  |  |  |  |
|  | 0.03 vs 0.04 | 0.03 | 1.78 | 0.48 |  |  |  |  |  |
|  | 0.025 (background medium vs 0.025 (rich medium) | 2.34 | 1.78 | 0.018 | 5.50 | 4.74 | | | 0.03 |

Supplementary Table 2. Analysis of variance (one-way ANOVA) and T-test with equal variance of the Cell Dry Weight (CDW) and % of glucans of *L. edodes* biomass, cultivated in stirred tank reactor (STR) by varying by varying the dilution rate of semicontinuous fermentations. Grey highlighted values are the statistically significant values. (when P-value < 0.05)
